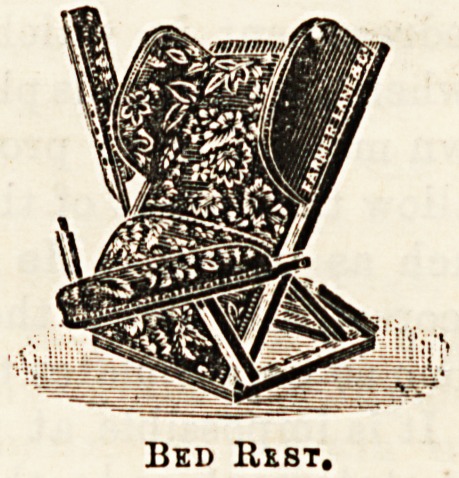# New Appliances and Things Medical

**Published:** 1898-10-08

**Authors:** 


### NEW APPLIANCES AND THINGS MEDICAL.

[We shall be glad to receive, at our Office, 28 & 29, Southampton Street, Strand, London, W.C., from the manufacturers, specimens of all new preparations and appliances which may be brought out from time to time.]

### ERGOT ASEPTIC.

(Parke, Davis, and Co., 20, North Audley Street, London, W.)

This is a preparation of ergot made in such a manner and put up in such a form as to be thoroughly aseptic, and thus to be fitted for use as a hypodermic medication without any fear of injurious consequences. It is supplied in hermetically sealed glass bulbs, each of which contains an ordinary dose. On the shank of the bulb there is a mark where it is to be broken off, and when this is done we have at our disposal, without any further preparation, the dose which is likely to be required. The point of the needle can then be inserted into the liquid in the bulb, and so charged with the aseptic ergot. It is stated that this preparation is quite free from ergotinic acid and other injurious parts of the ergot, and that it also contains no preservative, being merely sterilised and sealed up. Its uniform activity is guaranteed by each parcel being standardised. There is no doubt about the utility of this preparation.

### PROTARGOL

(Farbenfabriken vorm. Friedr. Bayer and Co., Elberfeld.)

Protargol is the name given to a preparation of silver in which the metal is so combined with protein substances that it is no longer precipitated by contact with albumen or sodium chloride. Herein lies its value, for it gives us a clear solution of a silver salt which will remain unchanged, and therefore will keep such activity as it possesses even after contact with living membranes. The utility of this attribute when the preparation is used as an injection is obvious. The question then remains whether the preparation is an active one, for it is clear that a chemical compound might be so stable as to be without effect on the mucous membrane. This, however, does not seem to be the case, for its effects when applied in cases of urethritis have been good. As an injection it is to be used in the strength of ½ to 1 per cent. for the posterior portion of the canal, and up to 3 per cent. for anterior urethritis.

### MAGGI'S CONSOMME FOR HOSPITAL USE.

(London Agents: Messrs. Cosenza and Co., 95, Wigmore Street.)

We may again draw attention to the preparation in bulk of Maggi's Consommé for hospital use, for which we learn that there is a great demand. It is evidently a very cheap form of concentrated food. When prepared according to the directions, it makes an appetising clear soup, quite suitable for those of fastidious taste, and is likely to be of much use in hospitals and other institutions where sudden demands for such preparations have to be met. It is supplied in blocks of 1 lb., at 10s. per block, the soup costing less than a penny per cup of half a pint.

### INVALID FURNITURE AT MESSRS. FARMER, LANE, AND CO.'S.

A visit to Messrs. Farmer, Lane, and Co.'s establishment at 77, New Oxford Street, may truly be described as a liberal education. The number, variety, and excellence of all their goods excite the admiration, while the ingenuity displayed in anticipating an invalid's requirements renders his lot, if not exactly to be envied, at least one of comfortable endurance. We all recognise in illness, especially that of a chronic nature, how enormously the clever appliances of late years have helped to ameliorate its discomforts, and the question of what to get and the best place at which to get it is a constantly recurring one. Messrs. Farmer, Lane, and Co. have devoted much time and thought to all these matters with the result that they have brought all invalid and sick-room requisites to the highest pitch of perfection. On a recent visit to their show-rooms we were specially struck by the splendid quality of their chairs and reclining couches. We do not hesitate to say that their "Merlin" chairs stand unrivalled both for lightness, strength, and elegance of shape. One model is self-propelling as well as reclining, and is provided with an adjustable leg-rest, arms, and back. It has the further advantage of being perfectly noiseless in action. The price of this luxurious invention is £7 7s., which is very moderate. The "Hospital" chair is another useful variety, and should be in every institution which has the welfare of its patients at heart. Its action is self-propelling, and it is well and strongly made. Another admirable model of the same class at £2 10s. on wheels can easily be controlled by a person pushing from behind. Especially worthy of notice is a carrying chair of which we give an illustration; a lighter variety in bamboo is adapted for home use and takes up but little room. The "Combination" couch is another ingenious contrivance; it is adjustable at any angle, and packs up flat for travelling. Another and more elaborate variety is the "Improved Ilkley" couch. It is provided with cushions, which can be used or not as required, and is an ideal of comfort. Bed rests are another specialité. The illustration shows a very convenient design, which, being provided with arms, greatly adds to the comfort of the patient. The bed-table

Bed Table.

Carrying Chair.

Bed Rest.

shown in illustration is one that will meet a long-felt want, as it can be used equally well as a reading desk Bath chairs are another article for which this firm is justly famed. They are made either for propulsion by hand or for a small pony or donkey. Water beds and other appliances are always kept in stock, and can be lent on hire, which is a convenience that is greatly to be appreciated. The needs of our little ones have also been attended to, as shown by the splendid assortment of perambulators, bassinettes, and high table chairs, one of the latter having recently been supplied by Messrs. Farmer, Lane, and Co. for the infant daughter of the Czar.

### CYTOS BREAD.

(W. Marshall and Sons, Grimsby.)

The now well-known Cytos bread is a speciality of the firm of Marshall and Sons, of Grimsby. It is manufactured from the Cytos meal, a flour of peculiar excellence, and so prepared that it contains the germs of the grain, imparting to it nourishing and bone-forming qualities, which are frequently absent in flours of a high degree of refinement. The bread itself is of first-rate quality, and to be recommended not only for general household use, but as a delicacy for afternoon teas.

## Figures and Tables

**Figure f1:**
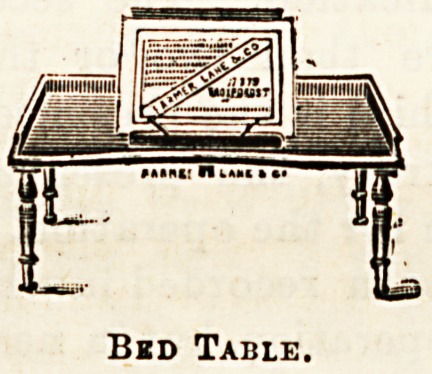


**Figure f2:**
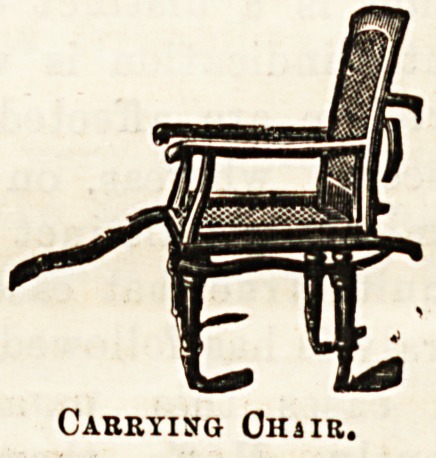


**Figure f3:**